# Variation in rates of post-operative oncological treatment for patients with glioblastoma in England: a comprehensive multi-year National cohort study from the GlioCova project

**DOI:** 10.1007/s11060-026-05440-7

**Published:** 2026-04-25

**Authors:** Radvile Mauricaite, Kerlann Le Calvez, Stephen David Robinson, Sophie Camp, Andrew Brodbelt, Thomas C. Booth, Matt Williams

**Affiliations:** 1 Department of Radiotherapy, Charing Cross Hospital, Imperial College Healthcare NHS Trust, Fulham Palace Road, W6 8RF, London, UK; 2 Computational Oncology Laboratory, Institute of Global Health Innovation, Imperial College London, London, UK; 3 Department of Neurosurgery, Charing Cross Hospital, Imperial College Healthcare NHS Trust, London, UK; 4 University Hospitals Sussex NHS Foundation Trust, Sussex Cancer Centre, Brighton, UK; 5 University of Sussex, Department of Biochemistry and Biomedicine, Falmer, Brighton, UK; 6 The Walton Centre NHS Foundations Trust, Liverpool, UK; 7 University of Liverpool, Liverpool, UK; 8 Department of Neuroradiology, King’s College Hospital NHS Foundation Trust, London, UK; 9 School of Biomedical Engineering & Imaging Sciences, King’s College London, London, UK

**Keywords:** Variation, Neurosurgery, Glioblastoma, Oncology

## Abstract

**Purpose:**

Although there are consistent guidelines of standardised glioblastoma patient care, there may be variation in treatments delivered due to differences in patient characteristics or adherence to guidelines across centres. This study explores the variation in treatments for glioblastoma patients across centres in a comprehensive multi-year brain tumour patient cohort in England.

**Methods:**

We analysed data from the Gliocova project that contains over 50,000 adult brain tumour patients diagnosed between 2013 and 2018 in England. We selected a glioblastoma patient cohort and explored treatment rates, systemic predictors of treatment, variation in treatment across surgical centres and survival rates for different treatment groups.

**Results:**

We analysed data from 11 359 patients with a histological diagnosis of GBM. Almost 80% of glioblastoma patients received at least some treatment after brain surgery, with 40% receiving the guideline recommended aggressive treatment (47% in the under-70s cohort). Age, sex, deprivation status, comorbidities, surgery type (resection versus biopsy) and ethnicity were identified as systemic predictors of receipt of post-operative treatment. There was also variation in receipt of post-operative treatment (64%-86%) and receipt of aggressive treatment (16%-65%) across centres. Patients receiving aggressive treatment had highest survival (15.6 months); in patients receiving any post operative treatment survival was 12.5 months and in patients receiving surgery only survival was 2.5 months.

**Conclusion:**

22% of glioblastoma patients undergoing surgery receive no further treatment and there is variation in post-surgical treatment both at centre and individual patient level. We suggest that centres should measure and report rates of no further treatment as part of standard clinical governance.

**Supplementary Information:**

The online version contains supplementary material available at 10.1007/s11060-026-05440-7.

## Introduction

Although brain tumours are rare, they significantly affect life expectancy and quality [1, 2]. The commonest malignant brain tumour in adults is glioblastoma which has the highest total years of life lost (YLL), with a mean of 19.8 YLL [3].

The standard of care first line treatment of glioblastoma includes maximal safe surgical debulking, followed by chemoradiotherapy with concurrent and adjuvant temozolomide [4–6]. This has remained the standard of care over the last 20 years, and is the basis for guidelines worldwide [7]. However, this treatment is only suitable for patients who are well enough to tolerate such aggressive treatment, and many patients receive less intensive treatment.

Internationally, there is limited work on variation in rates of post-operative oncology treatment and care in patients with glioblastoma. There is some work on variation in patterns of care in a region in Australia, in peri-operative steroid use in the USA, in patterns of post-operative imaging in glioblastoma in the UK, in surgical volumes in the UK and US, and some old work on use of radiotherapy [8–14]. There is one Norwegian study that looked at the differences in use of post-operative radiotherapy and temozolomide [15]. Most of the literature focuses on improving outcomes in the 1/3rd of patients who have aggressive treatment, but we know that improving outcomes in those who have less intensive treatment is important to patients [16].

We have previously demonstrated a clear difference in survival outcomes based on intensity of post-operative treatment; survival in those receiving surgery alone was 2 months; in those receiving intermediate treatment was 9 months; in those receiving aggressive treatment was 16 months [17]. Given that fewer than 1/3rd of patients receive aggressive oncological treatment, and the clear relationship between intensity of oncological treatment and survival, we examined the factors that influence receipt of post-operative treatment. In this paper, we explore variation in rates of receipt of post-operative treatment for newly diagnosed patients with glioblastoma in England. We explore rates of treatment vs. no treatment as well as aggressive treatment vs. intermediate treatment and look for evidence of systematic predictors of treatment and variation in practice between centres.

## Methods

### Dataset

The Gliocova dataset consists of data from all adults diagnosed with a brain tumour in England between 2013 and 2018. Data was extracted in August 2020 with mortality data (for survival) censored in October 2022. It contains data on over 50,000 brain tumour patients with linked data on demographics, surgery, chemotherapy, radiotherapy, hospital admissions, imaging and death.

### Patient cohort

We identified all patients with a newly diagnosed glioblastoma based on ICD-10 diagnosis code and tumour morphology (see Appendix for all the codes used in analyses). We excluded patients with more than one brain tumour diagnosis diagnosed at different time points, as we cannot be certain which treatments were related to which diagnosis. We identified patients undergoing a resection or biopsy based on OPCS codes as in previous work using HES data on inpatient admissions (See Appendix Table 3 and Table 4 for the codes used) [17]. We selected only the first surgical admission for these patients. If patients had a resection within 30-days of biopsy, we counted the resection admission as their first intervention admission. For radiotherapy and chemotherapy, we only selected treatments to the brain or spinal cord (See Appendix for the codes used).

### Treatments

In line with previous work, we identified three cohorts. We classified treatments as ‘Aggressive’, ‘Intermediate’ or ‘No post-operative’ treatment after surgery (either resection or biopsy) where ‘Aggressive’ treatment aligns with chemo-radiotherapy. Radical radiotherapy was defined as any treatment of equal or more than 40 Gy, and ‘Aggressive’ treatment was defined as surgery followed by radical radiotherapy (within 90 days of surgery) and at least one cycle of chemotherapy or trial drug. ‘Intermediate’ treatment was any combination of radiotherapy or chemotherapy that did not meet the definition for ‘Aggressive’ treatment. ‘No post-operative’ treatment was defined as those patients who had a histological diagnosis of a brain tumour (via biopsy/resection) but had no further radiotherapy or chemotherapy treatment. For this analysis, we excluded patients who did not have a histological diagnosis.

### Outcome measures

We explored rates of post-operative treatments. We calculated rates of post-operative treatment in two binary models. In the first, we explored rates of ‘Aggressive’ vs. ‘Intermediate’ and ‘No post-operative’ treatment. In the second, we explored rates of ‘Any post-operative’ treatment’ vs. ‘No post-operative’. Since our condition for treatment was histological diagnosis, we considered receipt of treatment based on surgical centre. Centre size was calculated by summing all cases over the six-year period for each surgical centre.

We calculated the Elixhauer Comorbidity Index score based on the diagnosis codes from the surgery admission using our previously defined modified Gliocova based Elixhauer Comorbidity Index weights (See Online Resource 1 and 2 for the codes used and the adjusted weights).

We explored systematic predictors of treatment by considering age, sex, ethnicity, co-morbidities, treatment type (resection versus biopsy) and deprivation status on the outcome of interest in univariable and multivariable models.

We depicted variation in receipt of treatment by centre using stacked barplots, and by funnel plots adjusted for overdispersion (if present). We explored survival in all three groups, calculating survival from the date of diagnosis to the date of death or censoring. Since age is known to be an important predictor of both survival and further treatment, we explored measures in the entire population, as well as in the those aged 18–69. To assess the impact of treatment on outcomes, we explored 1 year, 2 year and overall survival from diagnosis, and related that with intensity of oncological treatment.

### Statistical approaches

Binary logistic regression was performed in R (v 4.4.3) using glm function [18, 19]. We constructed models using a step-forward/backward approach using Akaike Information Criteria (AIC) as criteria to retain factors. Age and sex were included in the models even if eliminated during AIC elimination due to their a priori importance.

Risk-adjusted funnel plots were produced in R using FunnelPlotR package [20]. Predictions were obtained from a binary logistic regression model that adjusted for age, sex, ethnicity, deprivation status, comorbidities and surgery type (resection versus biopsy). Overdispersion was addressed using the Summary Hospital-level Mortality Indicator (SHMI) method embedded in the funnel_plot function. Crude funnel plots were based on observed proportions of treatment by centre without risk adjustment. Confidence limits (95% and 99.8%) were calculated using the fundata function from the FunnelPLotR package. Benchmark rate was defined as the overall pooled proportion across all centres.

Survival was calculated using the Kaplan-Meier method. P-values for the difference between groups were calculated using multivariate log-rank tests. Survival was defined as the time from diagnosis to death from any cause.

## Results

### Patient cohort and outcomes

There were 15,294 patients with a glioblastoma diagnosis of whom 15,181 had a single brain tumour diagnosis. Of these, 11,383 (75%) underwent tissue sampling. We removed 18 patients that had inconsistent admission information (e.g. same admission episode but multiple different intervals from diagnosis to that admission). We removed 6 patients where the data incorrectly identified surgical treatment centre leaving a final analytical cohort of 11,359 patients. The median age in the analytical cohort was 63 (IQR:54–70), median age varied across centres between 61 and 66 years and 62% (*N* = 6,990) were male. Median survival was 9.8 months (1-year OS: 42%; 2-year OS: 17%). 8,474 (75%) patients were under 70 years old. In this cohort the median age was 59 (IQR:51–65), 62% (*N* = 5,219) were male and median survival was 11.6 months (1-year OS: 48.5%; 2-year OS: 20%). For the distribution of age across centres see Appendix Fig. 4.

### Variation in care

78% (*N* = 8,797) of patients with a histological diagnosis received some post-operative treatment; 40% (*N* = 4,492) received ‘Aggressive’ treatment. There was significant variation in receipt of post-operative treatment by age and type of surgery and there was some variation by deprivation, sex and ethnicity (Table 1). In the under 70 cohort (*N* = 8,474), 81.7% (*N* = 6,922) patients received some post-operative treatment and 47% (*N* = 4,017) of patients received ‘Aggressive’ treatment.

**Table 1:** Variation in receipt of ‘Any post-operative’ and ‘Aggressive’ treatment by patient demographics

Category	Group	Total number of patients	Proportion of patients receiving ‘Any post-operative’ treatment	Proportion of patients receiving ‘Aggressive’ treatment
Age (5-year categories)	18–19	26	**0.88**	**0.46**
	20–24	54	**0.83**	**0.39**
	25–29	119	**0.87**	**0.55**
	30–34	196	**0.86**	**0.58**
	35–39	247	**0.84**	**0.49**
	40–44	432	**0.86**	**0.54**
	45–49	735	**0.87**	**0.53**
	50–54	1,132	**0.85**	**0.52**
	55–59	1,480	**0.82**	**0.50**
	60–64	1,811	**0.82**	**0.45**
	65–69	2,242	**0.76**	**0.40**
	70–74	1,721	**0.68**	**0.22**
	75–79	907	**0.63**	**0.10**
	80–84	223	**0.55**	**0.04**
	85–89	31	**0.29**	**0.00**
	90+	3	**0.00**	**0.00**
Sex	Male	6,990	**0.79**	**0.40**
	Female	4,369	**0.75**	**0.39**
Comorbidities	No comorbidities	6,291	**0.81**	**0.45**
	At least 1 comorbidity	5,068	**0.73**	**0.33**
IMD deprivation status (2015)	1 – Least deprived	2,777	**0.80**	**0.43**
	2	2,681	**0.78**	**0.39**
	3	2,277	**0.77**	**0.37**
	4	1,956	**0.75**	**0.40**
	5 – Most deprived	1,668	**0.77**	**0.38**
Treatment Type	Biopsy	3,410	**0.64**	**0.25**
	Resection	7,949	**0.83**	**0.46**
Ethnicity	Asian or Asian British	331	**0.74**	**0.37**
	Black or Black British	138	**0.75**	**0.42**
	Mixed	34	**0.71**	**0.44**
	Other Ethnic Groups	204	**0.78**	**0.48**
	Unknown / Not Stated	240	**0.70**	**0.36**
	White	10,412	**0.78**	**0.39**

On univariate analysis, increasing age, female sex, higher deprivation, Unknown ethnicity, higher co-morbidity scores and biopsy (vs. resection) all increased the chance of not receiving post-operative treatment. On multi-variable analysis, increasing age, female sex, increasing deprivation score, biopsy (vs. resection) and some ethnicity groups (Asian, Asian British or Unknown vs. White British) all increased the risk of not receiving post-operative treatment (Table 2).

**Table 2:** Logistic regression results for predicting receipt of post-operative treatment (univariate and multivariable models). Signif. Codes: 0 ‘***’ 0.001 ‘**’ 0.01 ‘*’ 0.05. Age and elixhauer comorbidity index were entered as continuous variables in both univariate and multivariable models

		Whole cohort (*N* = 11, 359)				Under 70s cohort (*N* = 8, 474)			
		Univariate		Multivariate		Univariate		Multivariate	
Variable	Categories	Odds Ratio (95% CI)	p-value	Odds Ratio (95% CI)	p-value	Odds Ratio (95% CI)	p-value	Odds Ratio (95% CI)	p-value
AGE (per 1 year increase)		0.96 (0.96–0.97)	< 2.00e^− 16^ ***	0.96 (0.96–0.97)	< 2.00e^− 16^ ***	0.98 (0.97–0.98)	< 0.001 ***	0.98 (0.97–0.99)	4.28e^− 12^ ***
SEX (Female)		0.81 (0.74–0.89)	5.49e^− 06^ ***	0.79 (0.72–0.86)	4.62e^− 07^ ***	0.86 (0.77–0.96	0.007 **	0.83 (0.74–0.94)	0.002**
Elixhauer Comorbidity Index score (total score; per 1-point increase)		0.86 (0.83–0.89)	3.7e^− 15^ ***	0.91 (0.87–0.94)	1.62e^− 07^ ***	0.91 (0.87–0.95)	< 0.001 ***	0.94 (0.90–0.98)	0.006**
IMD deprivation quintiles (2015)	**1- Least deprived (ref.)**	**-**	**-**	**-**	**-**	**-**	**-**	**-**	**-**
	**2**	0.88 (0.78-1.00)	0.049 *	0.87 (0.76–1.00)	0.048*	0.85 (0.72–1.01)	0.061	0.85 (0.72–1.01)	0.062
	**3**	0.84 (0.73–0.96)	0.001 **	0.84 (0.73–0.97)	0.016 *	0.83 (0.70–0.98)	0.031 *	0.83 (0.70–1.00)	0.046 *
	**4**	0.78 (0.68–0.90)	< 0.001 ***	0.74 (0.64–0.86)	5.69e^− 05^ ***	0.72 (0.61–0.86)	< 0.001 ***	0.74 (0.62–0.88)	0.001 ***
	**5- Most deprived**	0.84 (0.73–0.98)	0.021 *	0.73 (0.63–0.86)	8.89e^− 05^ ***	0.77 (0.64–0.92)	0.005 **	0.73 (0.60–0.88)	0.001 ***
Surgery type (resection)		2.81 (2.56–3.08)	< 2.00e^− 16^ ***	2.63 (2.40–2.89)	< 2.00e^− 16^ ***	2.99 (2.67–3.35)	< 0.001 ***	2.90 (2.58–3.25)	< 2.00e^− 16^ ***
Ethnicity	**White (ref.)**	-	-	-	-	-	-	-	-
	**Asian or Asian British**	0.81 (0.64–1.05)	0.109	0.71 (0.55–0.94)	0.013 *	0.81 (0.61–1.10)	0.164	0.76 (0.57–1.04)	0.081
	**Black or Black British**	0.84 (0.58–1.26)	0.382	0.72 (0.48–1.09)	0.107	0.72 (0.47–1.13)	0.135	0.68 (0.44–1.09)	0.100
	**Mixed**	0.69 (0.34–1.51)	0.319	0.62 (0.29–1.43)	0.241	0.68 (0.31–1.73)	0.384	0.70 (0.30–1.82)	0.425
	**Other Ethnic Groups**	1.04 (0.75–1.47)	0.818	0.90 (0.63–1.29)	0.539	0.94 (0.65–1.40)	0.740	0.89 (0.61–1.34)	0.569
	**Unknown / Not stated**	0.68 (0.52–0.91)	0.007 **	0.66 (0.49–0.89)	0.005 **	0.60 (0.44–0.83)	0.002 **	0.64 (0.46–0.90)	0.009 **

**Fig. 1:**
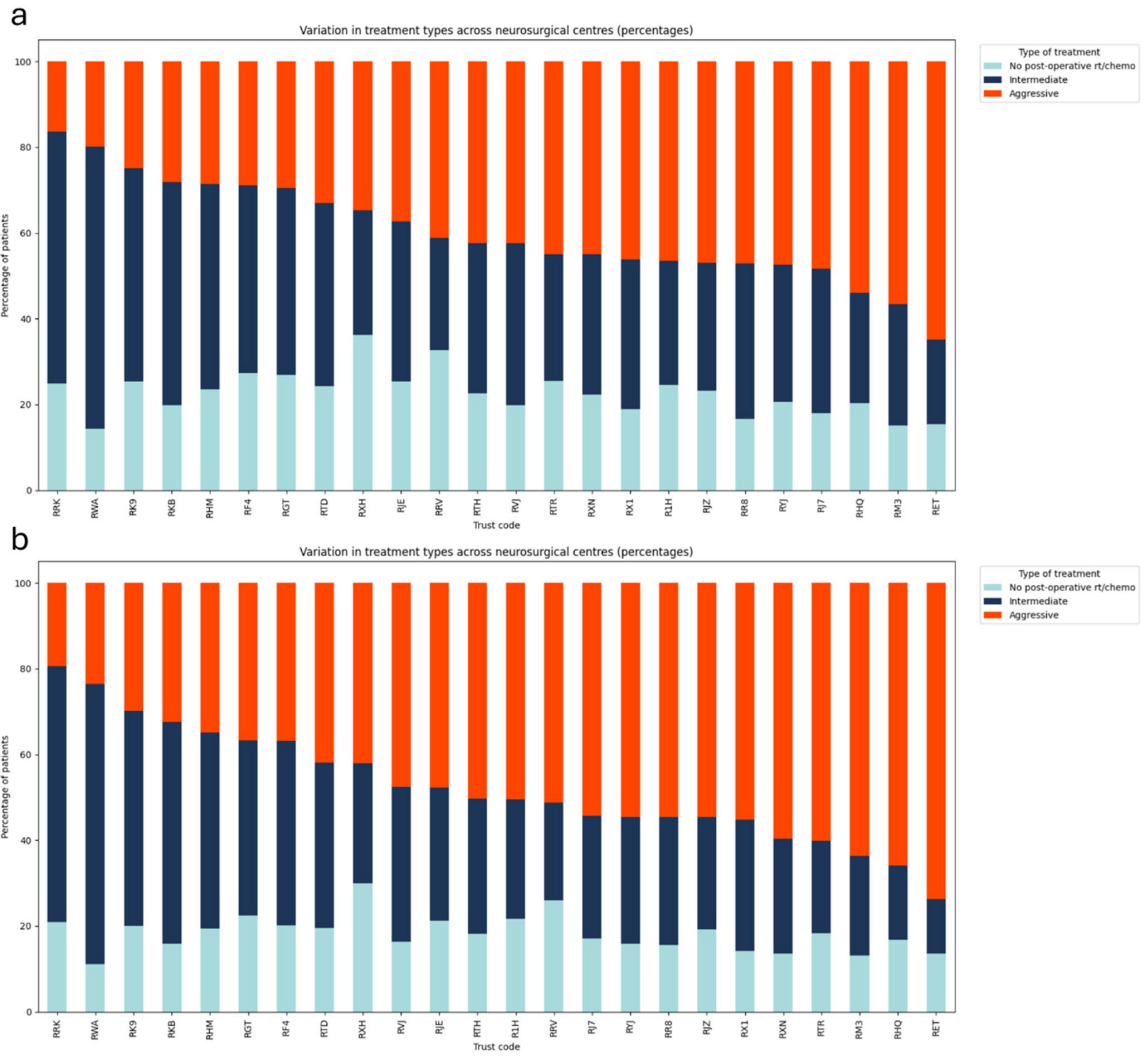
Stacked bar charts of the proportion of patients receiving ‘No post-operative’, ‘Intermediate’ and ‘Aggressive’ treatment by surgical centre in (**a**) the whole cohort (**b**) the under 70s cohort

At a centre level, there was significant variation in treatment rates between centres, with between 64% and 86% of patients receiving post-operative treatment, depending on where they had their surgery. There was greater variation in the type of treatment received across centres with numbers varying between 16% and 65% of patients receiving aggressive treatment for their glioblastoma. In the under 70s cohort, post-operative treatment rates varied from 70% to 89% and receipt of aggressive treatment rates varied from 19% to 74% between centres. Figure 1 shows stacked bar graphs for post-operative treatment.

**Fig. 2:**
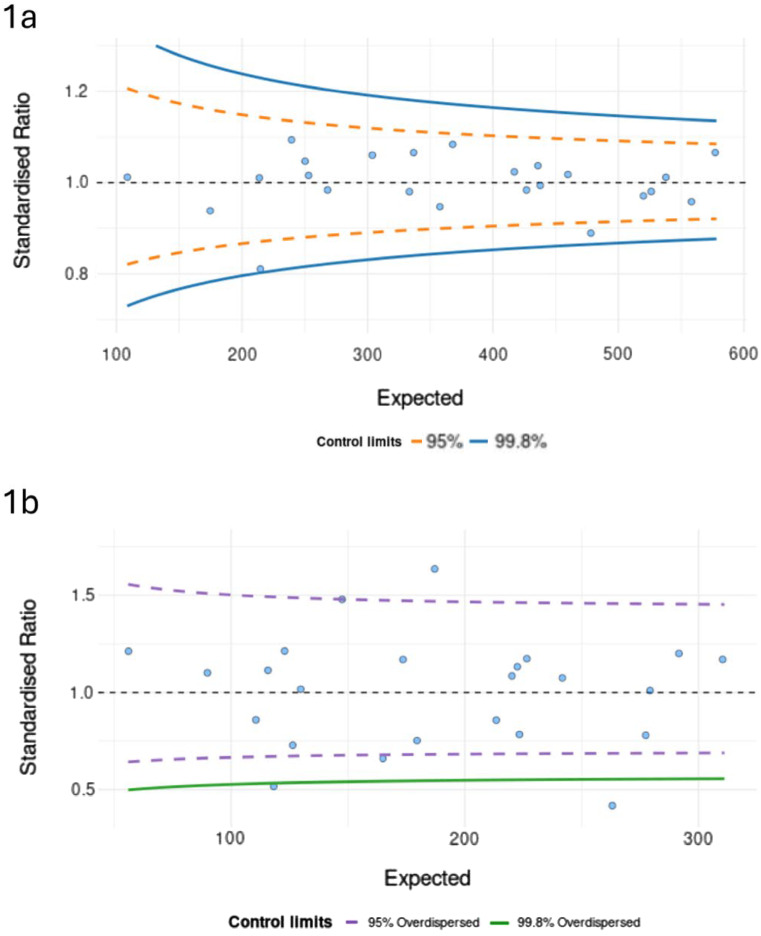

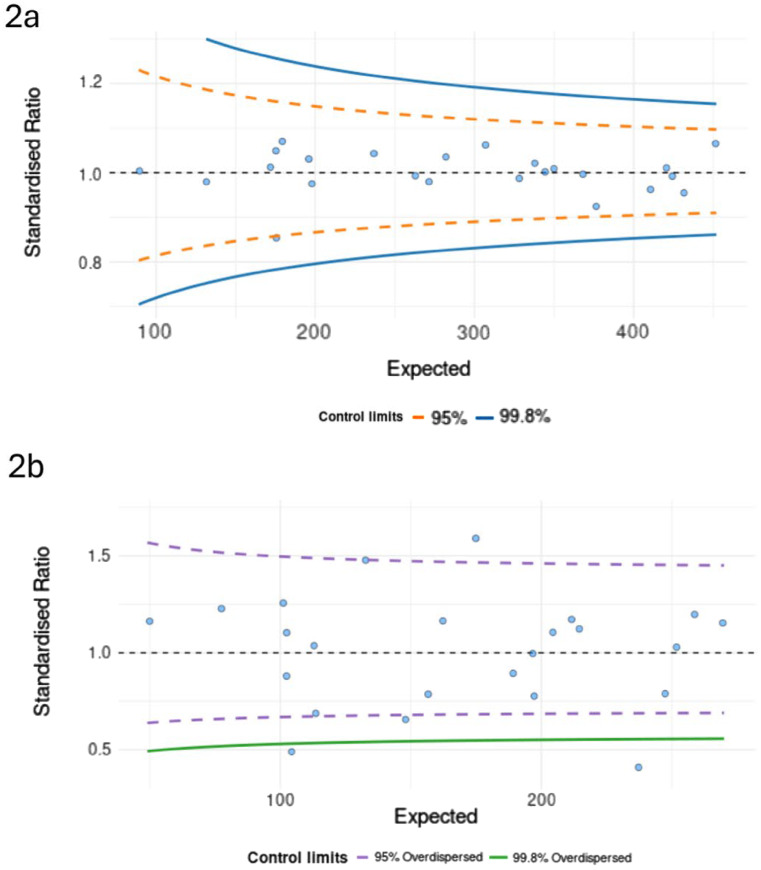
Risk-adjusted funnel plots showing variation in treatment in (1) whole cohort, (2) under 70s cohort, adjusted for over-dispersion where appropriate. Each centre is a single point, and the graphs show variation by the expected numbers of patients per trust with 95% and 99.8% control limits. Standardised ratio is the ratio between the observed and the expected (model predicted) outcome values, with SR = 1 denoting that the outcomes in a selected hospital are exactly as expected based on its patient population. **a** Variation in receipt of any post-operative treatment by centre. There was no evidence of over-dispersion, and hence this is not adjusted for over-dispersion. **b** Variation in receipt of aggressive treatment by centre, adjusted for over-dispersion. Even adjusting for overdispersion, there remain outliers in receipt of aggressive treatment

To further investigate this variation in receipt of post-operative treatment, we used funnel plots to visualise the variation in receipt of (a) ‘Any post-operative’ treatment versus ‘No post-operative’ treatment and (b) ‘Aggressive’ versus ‘Intermediate’ treatment (See Fig. 2). We adjusted for age, sex, comorbidity score, ethnicity, treatment type and deprivation status (Crude funnel plots in Appendix Fig. 6).

We have also briefly looked at variation in 30-day mortality after first surgical intervention across neurosurgical centres (See Appendix Fig. 5).

### Survival

In line with previous work, median survival in those receiving surgery alone was 2.5 months (1-year OS: 7%; 2-year OS: 3%); in those receiving any post-surgical treatment was 12.5 months (1-year OS: 52%; 2-year OS: 20%); in those receiving ‘Aggressive’ treatment, survival was 15.6 months (1-year OS: 66%; 2-year OS: 28%); those receiving ‘Intermediate’ treatment was 9.4 months (1-year OS: 37%; 2-year OS: 13%). In the under 70s cohort, survival was 2.4 (1-year OS: 9%; 2-year OS: 5%) months for those receiving surgery alone and 13.8 months (1-year OS: 57%; 2-year OS: 24%) for those receiving any post-operative treatment, with 16.0 months (1-year OS: 68%; 2-year OS: 30%) for those having ‘Aggressive’ treatment and 10.5 months (1-year OS: 43%; 2-year OS: 16%) for those having ‘Intermediate’ treatment (Appendix Fig. 7 for survival curves).

**Fig. 3:**
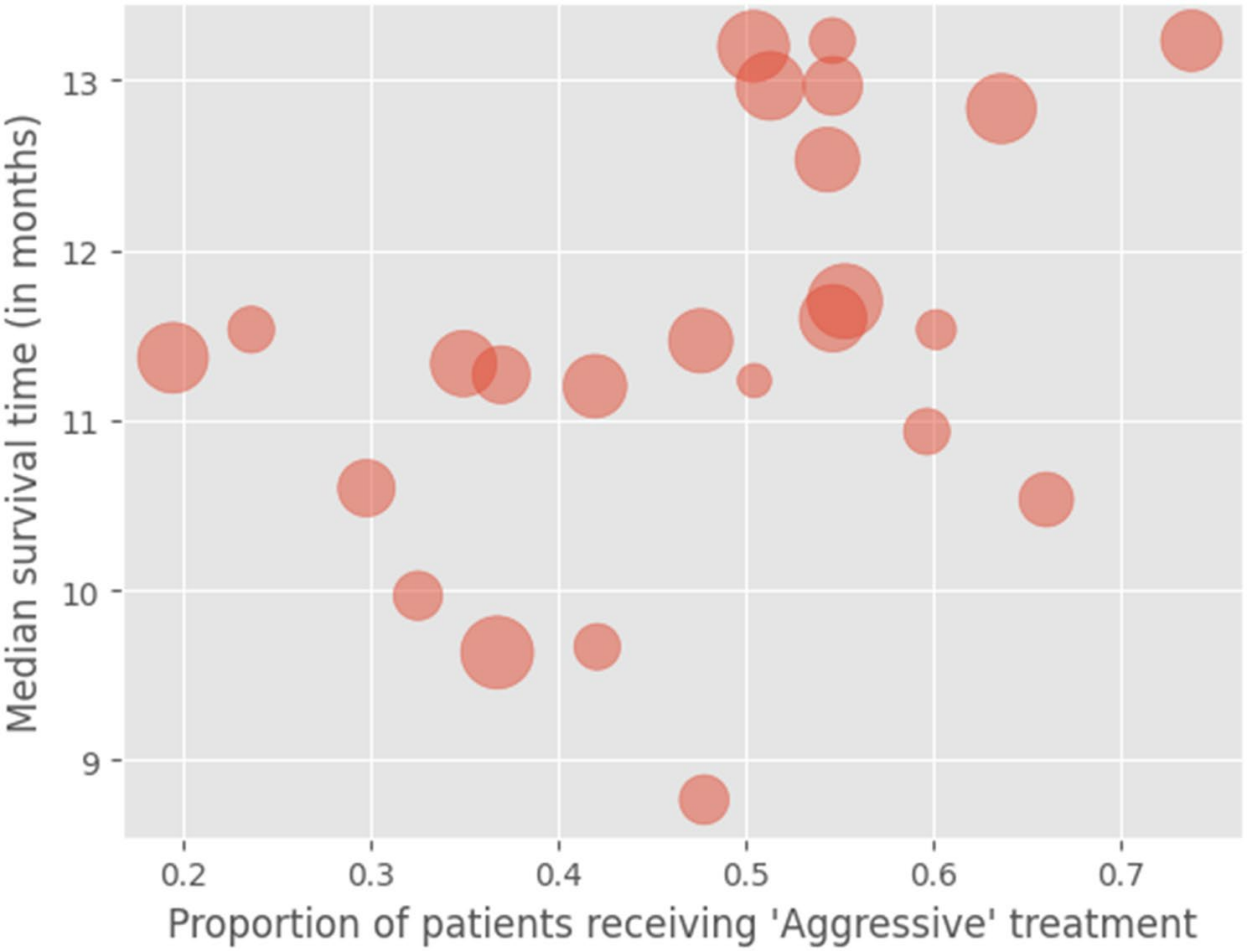
Variation in the proportion of patients treated ‘Aggressively’ and overall median survival for newly diagnosed glioblastoma patients under 70 across neurosurgical centres. Bubble size indicates centre volume – the total number of patients in each centre that received surgery

In the under-70s cohort of patients, median overall survival varied between centres from 8.8 to 13.2 months and proportion of patients receiving ‘Aggressive’ treatment varied from 0.2 to 0.7 between neurosurgical centres. See Fig. 3 below.

## Discussion

We have conducted the first ever national analysis of variation in receipt of post-operative treatment in patients with glioblastoma. Only 78% of patients received any post-operative oncological treatment, with 40% of patients receiving chemo-radiotherapy. Much of this is driven by patient factors, even in the under-70 cohort, adjusted for patient factors, there is striking variation in rates of treatment. The proportion of under-70s receiving any treatment varies between 70% to 89% and between 19% and 74% of patients receiving aggressive treatment. This variation is largely driven by variation in the delivery of ‘Aggressive’ versus ‘Intermediate’ treatment across centres, and two centres delivered significantly lower rates of ‘Aggressive’ treatments in risk adjusted models, even when adjusted for overdispersion.

Our findings on rates of treatment are broadly in line with other work. In a US study 45% of patients did not receive any temozolomide or radiotherapy [21] but this was based on a smaller cohort than ours (*N* = 2,272) and it only considered temozolomide. In an older English study of patients aged < 70, 29% of patients received no treatment or RT alone (minimal treatment) [10]; in a Korean study of over 3,000 glioblastoma patients over 14 years, 82% received concurrent chemoradiotherapy following surgery and in a Dutch study, 67% of newly diagnosed glioblastoma patients received adjuvant combined chemotherapy and radiotherapy post-operatively [22, 23].

Similarly, our findings of variation are in line with previous work. A previous English study showed regional variation with crude rates of surgery and chemotherapy lowest in London (19%) and highest in the North East of England [10]; a Norwegian study showed that fewer patients received chemotherapy and/or radiotherapy in the North, where the population was older, compared to other regions with the rate of post-operative radiotherapy varying from 80% to 94% and the rate of post-operative temozolomide administration varying between 71% and 92% [15].

Our data on patterns of surgical resection is also similar to previous work. The rate of biopsy vs. resection in our study is 30% versus 70% in the whole cohort and 28% versus 72% in the under 70s cohort. This is broadly in line with other international data: a national Dutch study of newly diagnosed glioblastoma patients showed that 32% of patients underwent a biopsy and 68% underwent resection [23], a US study showed that 56% of patients underwent resection [24] and in a Norwegian study rates of resection varied between 65 and 70% and > 90% depending on the region [15].

There are some weaknesses of our study. Firstly, we cannot explore reasons for non-receipt of treatment. Some patients will have been well pre-operatively and suffered surgical complications; such changes are difficult to predict. However, previous data on post-operative complications suggest that < 10% of patients have significant post-operative complications. Similarly, there are patients who need emergency surgery, but previous data suggest that numbers of such patients are very small (< 5/ year per centre). It is therefore difficult to believe that this explains most of the variation. Secondly, the dataset is now relatively old. Nonetheless, it provides evidence of variation at this time and is an important baseline for future work. There are some methodological considerations as well: centre size is defined only based on the number of glioblastoma patients; true neurosurgical volume might be better defined based on all primary and metastatic tumour surgery, although glioblastoma is the commonest adult malignant brain tumour, and makes up the majority of workload. In addition, there may be a secondary effect of the relationship between surgical and oncology treatment centres which might influence the rate of treatment, or time from surgery to oncology treatment, although previous (unpublished) work has not shown evidence of impact. Finally, we do not have data on the degree of resection, MGMT methylation status and performance status in our dataset, all of which could impact variation between centres.

The key finding of this study is that there is significant variation in rates and types of post-operative treatment for patients with glioblastoma in England. This is driven by both patient and centre level effects, and the majority of variation between centres is driven by differences in populations. However, there is significant group of patients who do not have post-operative treatment and have a very poor prognosis. We have previously shown that their prognosis is the same as those who have no surgery, and we would therefore argue that this represents futile care.

Informal discussion between centres in the UK has revealed substantial variation in the organisation of care. It is also important to note that there is a group of patients who receive oncology treatment without a tissue diagnosis and have significantly better survival than those who have a tissue diagnosis and no further treatment. The choice between these two routes is therefore an important choice to make, especially in older patients, and those who might only be offered a biopsy [17].

We suggest that centres should explicitly measure and report rates of no further treatment as part of standard clinical governance. There have been discussions about the need for a national brain tumour audit in England (Re: New cancer plan for England – The need for a National Brain Tumour Audit | The BMJ) and such measures could be included in this. There is also an open question as to how much of this is unique to England, and how much is a generalised finding across multiple countries. There is little good data to answer this question and is the subject of ongoing research.

## Appendix

### Codes used in the analyses

#### Glioblastoma patient cohort

Glioblastoma tumour was defined using the International Classification of Diseases 10th Revision diagnosis codes C70-C72, ICD-O-3 morphology codes 9440–9442 and WHO grades coded as G3, G4, GX, and field left blank. Glioblastoma patients coded with a grade 3, grade ‘X’ or with a field left blank had a similar survival to those with a grade 4 glioblastoma. We therefore assumed those patients had been miscoded as the data is historic and predates the new WHO recommendations.

#### Selected radiotherapy treatments

In radiotherapy, only primary diagnoses starting by C70-C72 were selected along with a primary region treated (‘P’, ‘R’, ‘PR’) or with procedure codes for brain or spine recorded (i.e., Z01, Z06, Z07, Z66, Z67).

#### Selection of resection and biopsy

**Table 3:** Selection of the OPCS codes to capture resections

Code	Label
A012	Total lobectomy of brain
A013	Partial lobectomy of brain
A018	Other specified major excision of tissue of brain
A019	Unspecified major excision of tissue of brain
A021	Excision of lesion of tissue of frontal lobe of brain
A022	Excision of lesion of tissue of temporal lobe of brain
A023	Excision of lesion of tissue of parietal lobe of brain
A024	Excision of lesion of tissue of occipital lobe of brain
A025	Excision of lesion of tissue of cerebellum
A026	Excision of lesion of tissue of brain stem
A027	Excision of transcranial dermoid cyst
A028	Other specified excision of lesion of tissue of brain
A029	Unspecified excision of lesion of tissue of brain
A073	Exploration of tissue of brain
A078	Other specified other open operations on tissue of brain
A108	Other specified other operations on tissue of brain
A118	Other specified operations on tissue of brain
A168	Other specified other open operations on ventricle of brain
A171	Endoscopic extirpation of lesion of ventricle of brain
A208	Other specified other operations on ventricle of brain
A291	Excision of lesion of optic nerve (ii)
A293	Excision of lesion of trigeminal nerve (v)
A295	Excision of lesion of acoustic nerve (viii)
A298	Excision of lesion of specified cranial nerve NEC
A381	Extirpation of lesion of meninges of cortex of brain
A382	Extirpation of lesion of meninges of sphenoidal ridge of cranium
A383	Extirpation of lesion of meninges of subfrontal region of brain
A384	Extirpation of lesion of meninges of parasagittal region of brain
A385	Extirpation of lesion of falx cerebri
A386	Extirpation of lesion of tentorium cerebelli
A388	Other specified extirpation of lesion of meninges of brain
A389	Unspecified extirpation of lesion of meninges of brain
A428	Other specified other operations on meninges of brain
A431	Extirpation of lesion of meninges of skull base
A432	Extirpation of lesion of meninges of skull clivus
A438	Other specified other extirpation of lesion of meninges of brain
A442	Extirpation of lesion of spinal cord NEC
A443	Excision of lesion of intradural intramedullary spinal cord
A444	Excision of lesion of extradural spinal cord
A445	Excision of lesion of intradural extramedullary spinal cord
A448	Other specified partial extirpation of spinal cord
A449	Unspecified partial extirpation of spinal cord
A511	Extirpation of lesion of meninges of spinal cord
A518	Other specified other operations on meninges of spinal cord
A571	Extirpation of lesion of spinal nerve root
A599	Unspecified excision of peripheral nerve
A611	Excision of lesion of peripheral nerve
B012	Trans-sphenoidal hypophysectomy
B068	Other specified operations on pineal gland
C021	Excision of lesion of orbit
E158	Other specified operations on sphenoid sinus
T962	Excision of lesion of soft tissue NEC
V031	Exploratory open craniotomy
V038	Other specified opening of cranium
V039	Unspecified opening of cranium
V051	Extirpation of lesion of cranium
V058	Other specified other operations on cranium
V431	Excision of lesion of cervical vertebra
V433	Excision of lesion of lumbar vertebra
V498	Other specified exploration of spine
V499	Unspecified exploration of spine
Y059	Unspecified excision of organ NOC
Y068	Other specified excision of lesion of organ NOC
Y069	Unspecified excision of lesion of organ NOC
Y461	Trans-sphenoidal open approach to contents of cranium
Y463	Transoral open approach to contents of cranium
Y464	Transmastoid open approach to contents of cranium
Y465	Supratentorial open approach to contents of cranium
Y467	Craniectomy approach to contents of cranium
Y468	Other specified open approach to contents of cranium
Y469	Unspecified open approach to contents of cranium
Y470	Trans-cranial approach to contents of cranium
Y471	Trans-sphenoidal burrhole approach to contents of cranium
Y472	Frontal burrhole approach to contents of cranium
Y473	Transoral burrhole approach to contents of cranium
Y474	Transmastoid burrhole approach to contents of cranium
Y475	Supratentorial burrhole approach to contents of cranium
Y476	Infratentorial burrhole approach to contents of cranium
Y478	Other specified burrhole approach to contents of cranium
Y479	Unspecified burrhole approach to contents of cranium
Y698	Other specified harvest of other tissue

**Table 4:** Selection of the OPCS codes to capture biopsies

Code	Label
A041	Open biopsy of lesion of tissue of frontal lobe of brain
A042	Open biopsy of lesion of tissue of temporal lobe of brain
A043	Open biopsy of lesion of tissue of parietal lobe of brain
A044	Open biopsy of lesion of tissue of occipital lobe of brain
A081	Biopsy of lesion of tissue of frontal lobe of brain NEC
A082	Biopsy of lesion of tissue of temporal lobe of brain NEC
A083	Biopsy of lesion of tissue of parietal lobe of brain NEC
A084	Biopsy of lesion of tissue of occipital lobe of brain NEC
A085	Biopsy of lesion of tissue of cerebellum NEC
A086	Biopsy of lesion of tissue of brain stem NEC
A088	Other specified other biopsy of lesion of tissue of brain
A089	Unspecified other biopsy of lesion of tissue of brain
A104	Aspiration of lesion of tissue of brain NEC
A105	Puncture of tissue of brain NEC
A181	Diagnostic endoscopic examination of ventricle of brain and biopsy of lesion of ventricle of brain
A188	Other specified diagnostic endoscopic examination of ventricle of brain
A363	Biopsy of lesion of cranial nerve
A422	Biopsy of lesion of meninges of brain
A454	Open biopsy of lesion of spinal cord
A456	Open aspiration of lesion of spinal cord
A481	Biopsy of lesion of spinal cord NEC
A482	Aspiration of lesion of spinal cord
A513	Biopsy of lesion of meninges of spinal cord
A578	Other specified operations on spinal nerve root
A731	Biopsy of lesion of peripheral nerve
B042	Biopsy of lesion of pituitary gland
T968	Other specified other operations on soft tissue
V036	Exploratory burrhole of cranium
V052	Biopsy of lesion of cranium
Y201	Stereotactic biopsy of lesion of organ NOC
Y202	Stereotactic biopsy of organ NOC
Y208	Other specified biopsy of organ NOC
Y462	Frontal open approach to contents of cranium
Y466	Infratentorial open approach to contents of cranium
Y471	Trans-sphenoidal burrhole approach to contents of cranium
Y472	Frontal burrhole approach to contents of cranium
Y473	Transoral burrhole approach to contents of cranium
Y474	Transmastoid burrhole approach to contents of cranium
Y475	Supratentorial burrhole approach to contents of cranium
Y476	Infratentorial burrhole approach to contents of cranium
Y478	Other specified burrhole approach to contents of cranium
Y479	Unspecified burrhole approach to contents of cranium
Y698	Other specified harvest of other tissue

## Complementary figures

**Fig. 4:**
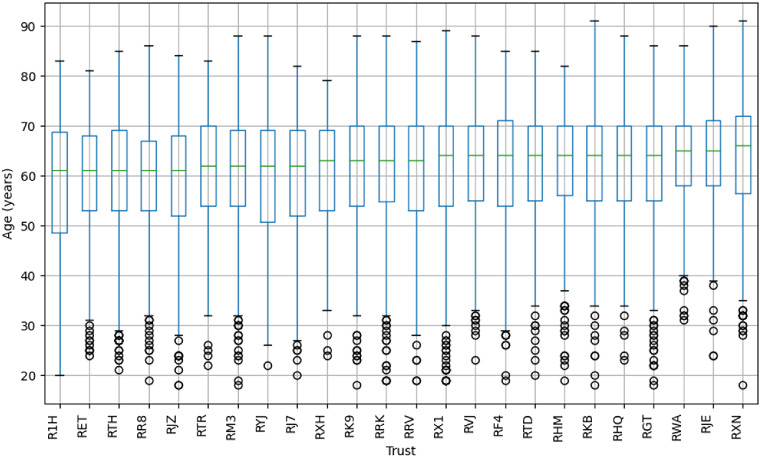
Boxplot showing the distribution of age (age at diagnosis) across neurosurgical centres with midline indicating median age

**Fig. 5:**
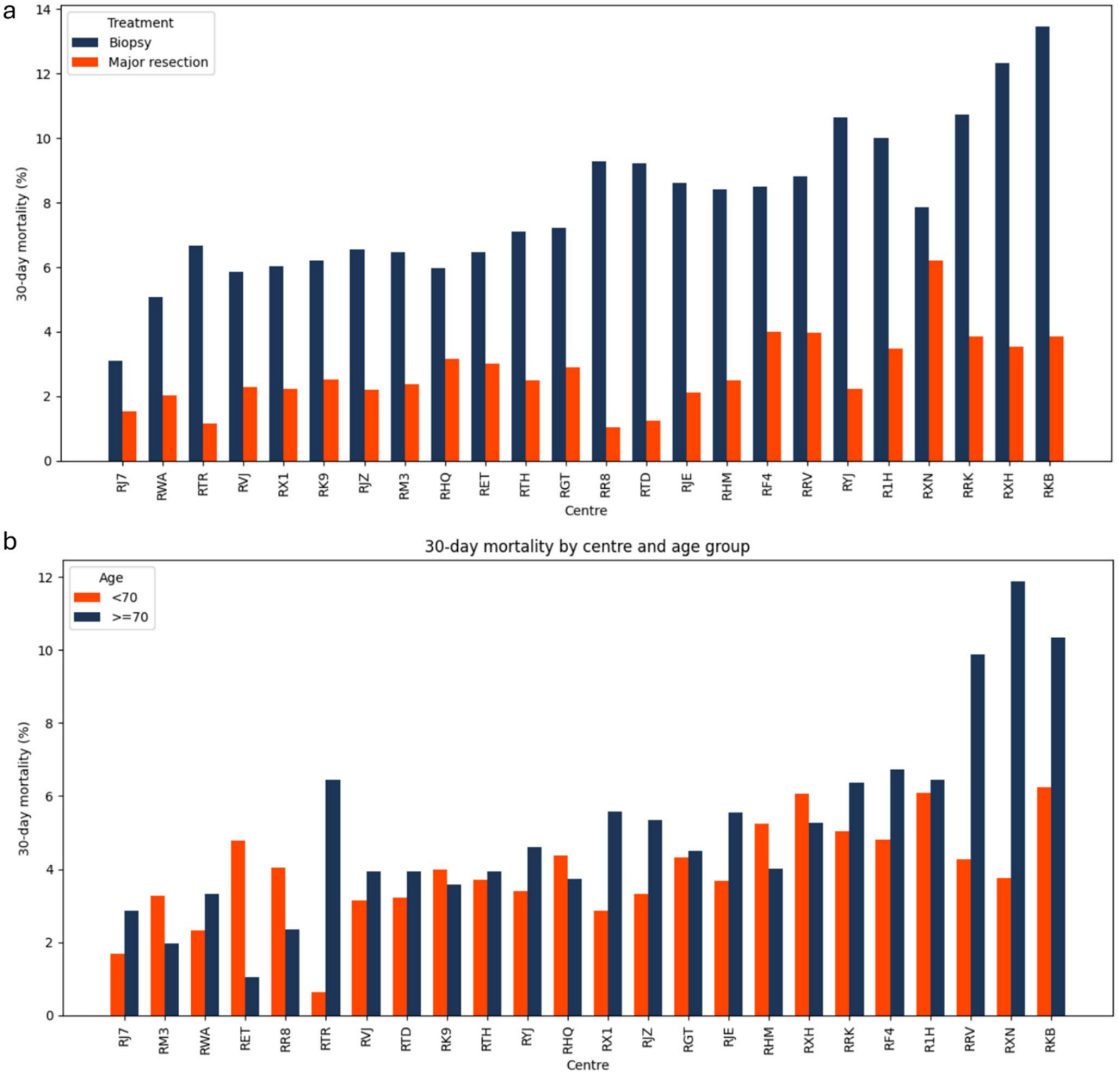
Variation in percentage 30-day mortality across neurosurgical centres by (**a**) treatment type and (**b**) age groups

**Fig. 6:**
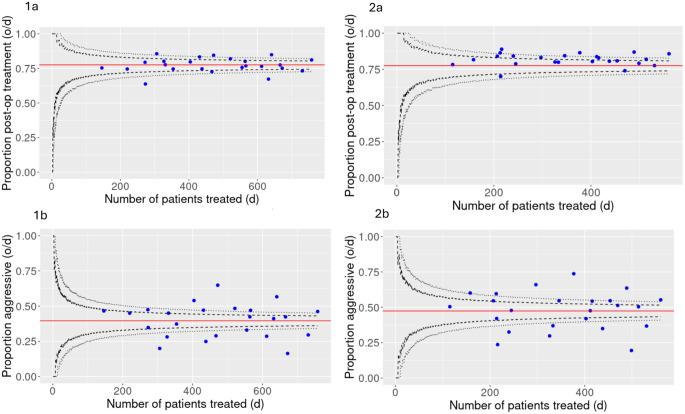
Funnel plots showing the observed proportion of patients (1) in the whole cohort and (2) in the under 70s cohort receiving (**a**) ‘Some post-operative’ treatment or (**b**) ‘Aggressive’ treatment across centres with the observed numbers of patients per centre. Each dot represents a centre with dashed lines denoting the 95% and 99.8% control limits

**Fig. 7:**
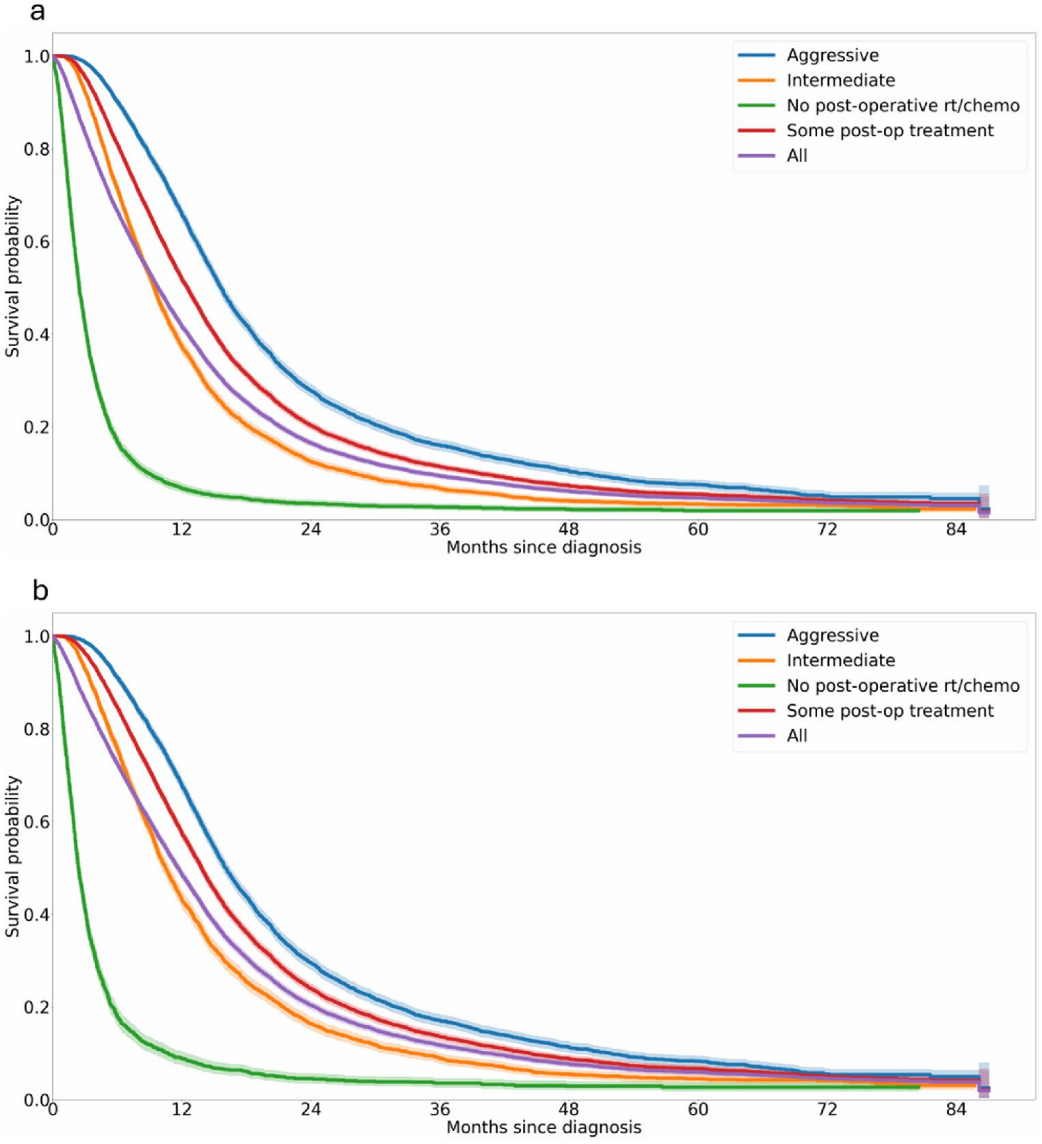
Survival curves showing overall survival of newly diagnosed glioblastoma patients from diagnosis to death or censoring based on different treatment types in (**a**) whole cohort and (**b**) under 70s cohort

## Supplementary Information

Below is the link to the electronic supplementary material.

Supplementary Material 1

Supplementary Material 2

## Data Availability

We are open to collaborating with research teams as we believe in sharing knowledge. If people are interested in working on Gliocova, they can contact us to enquire about data access. The data is securely held on Imperial College London servers so arrangements will need to be made prior to access.
