# Supplementary material for: Variation in rates of post-operative oncological treatment for patients with glioblastoma in England: a comprehensive multi-year National cohort study from the GlioCova project
Source: J Neurooncol. 2026 Apr 25;177(3):126. doi: 10.1007/s11060-026-05440-7 (PMC13110236; doi:10.1007/s11060-026-05440-7)
Supplement: Supplementary file 1 — Supplementary Material 1 [file 11060_2026_5440_MOESM1_ESM.docx]

**Article title:** Variation in rates of post-operative oncological treatment for patients with glioblastoma in England: A comprehensive multi-year national cohort study from the GlioCova project

**Journal:** Journal of Neuro-Oncology

**Authors:** Radvile Mauricaite (BSc), Kerlann Le Calvez (MSc), Stephen David Robinson (BMBS), Sophie Camp (PhD), Andy Brodbelt (PhD), Thomas C Booth (PhD) and Matt Williams (PhD)

**Corresponding author:** Dr. Matt Williams, Radiotherapy department, Charing Cross Hospital, Fulham Palace Road, W6 8RF, London, UK, [matthew.williams@imperial.ac.uk](mailto:matthew.williams@imperial.ac.uk)

# Online Resource 1

Elixhauer Comorbidity Index variables and the corresponding ICD-10 codes used to identify them

| **Comorbidity** | **ICD_10 codes used** |
| --- | --- |
| Congestive heart failure | I099 ,I110,I130,I132,I255,I420,I425,I426,I427,I428,I429,I43X,I50X,P290 |
| Cardiac arrhytmias | I441,I442,I443,I456,I459,I47X,I48X,I49X,R000,R001,R008,T821,Z450,Z950 |
| Valvular disease | A520,I05X,I06X,I07X,I08X,I091,I098,I34X,I35X,I36X,I37X,I38X,I39X,Q230,Q231,Q232,Q233,Z952,Z953,Z954 |
| Pulmonary circulation disorders | I26X,I27X,I280,I288,I289 |
| Peripheral vascular disorders | I70X,I71X,I731,I738,I739,I771,I790,I792,K551,K558,K559,Z958,Z959 |
| Hypertension, compicated | I11X,I12X,I13X,I15X |
| Hypertension, uncomplicated | I10X |
| Paralysis | G041,G114,G801,G802,G81X,G82X,G830,G831,G832,G833,G834,G839 |
| Other neurological disorders | G10X,G11X,G12X,G13X,G20X,G21X,G22X,G254,G255,G312,G318,G319,G32X,G35X,G36X,G37X,G40X,G41X,G931,G934,R470,R56X |
| Chronic pulmonary disease | I278,I279,J40X,J41X,J42X,J43X,J44X,J45X,J46X,J47X,J60X,J61X,J62X,J63X,J64X,J65X,J66X,J67X,J684,J701,J703 |
| Diabetes, uncomplicated | E100,E101,E109,E10X,E111,E119,E120,E121,E129,E130,E131,E139,E140,E141,E149 |
| Diabetes, complicated | E102,E103,E104,E105,E106,E107,E108,E112,E113,E114,E115,E116,E117,E118,E122,E123,E124,E125,E126,E127,E128,E132,E133,E134,E135,E136,E137,E138,E142,E143,E144,E145,E146,E147,E148 |
| Hypothyroidism | E00X,E01X,E02X,E03X,E890 |
| Renal failure | I120,I131,N18X,N19X,N250,Z490,Z491,Z492,Z940,Z992 |
| Liver disease | B18X,I85X,I864,I982,K70X,K711,K713,K714,K715,K717,K72X,K73X,K74X,K760,K762,K763,K764,K765,K766,K767,K768,K769,Z944 |
| Peptic ulcer disease excluding bleeding | K257,K259,K267,K269,K277,K279,K287,K289 |
| AIDS/HIV | B20X,B21X,B22X,B24X |
| Lymphoma | C81X,C82X,C83X,C84X,C85X,C88X,C96X,C900 |
| Metastatic cancer | C77X,C78X,C79X,C80X |
| Solid tumor without metastasis | C00X,C000,C001,C002,C003,C004,C005,C006,C008,C009,C01X,C02X,C020,C021,C022,C023,C024,C028,C029,C03X,C030,C031,C039,C04X,C040,C041,C048,C049,C05X,C050,C051,C052,C058,C059,C06X,C060,C061,C062,C068,C069,C07X,C08X,C080,C081,C088,C089,C09X,C090,C091,C098,C099,C10X,C100,C101,C102,C103,C104,C108,C109,C11X,C110,C111,C112,C113,C118,C119,C12X,C13X,C13,C131,C132,C138,C139,C14X,C140,C142,C148,C15X,C150,C151,C152,C153,C154,C155,C158,C159,C16X,C160,C161,C162,C163,C164,C165,C166,C168,C169,C17X,C170,C171,C172,C173,C178,C179,C18X,C180,C181,C182,C183,C184,C185,C186,C187,C188,C189,C19X,C20X,C21X,C210,C211,C212,C218,C22X,C220,C221,C222,C223,C224,C227,C229,C23X,C24X,C240,C241,C248,C249,C25X,C250,C251,C252,C253,C254,C257,C258,C259,C26X,C260,C261,C268,C269,C30X,C300,C301,C31X,C310,C311,C312,C313,C318,C319,C32X,C320,C321,C322,C323,C328,C329,C33X,C34X,C340,C341,C342,C343,C348,C349,C37X,C38X,C380,C381,C382,C383,C384,C388,C39X,C390,C398,C399,C40X,C400,C401,C40.2,C403,C408,C409,C41X,C410,C411,C412,C413,C414,C418,C419,C43X,C430,C431,C432,C433,C434,C435,C436,C437,C438,C439,C45X,C450,C451,C452,C457,C459,C46X,C460,C461,C462,C463,C467,C468,C469,C47X,C470,C471,C472,C473,C474,C475,C476,C478,C479,C48X,C480,C481,C482,C488,C49X,C490,C491,C492,C493,C494,C495,C496,C498,C499,C50X,C500,C501,C502,C503,C504,C505,C506,C508,C509,C51X,C510,C511,C512,C518,C519,C52X,C53X,C530,C531,C538,C539,C54X,C540,C541,C542,C543,C548,C549,C55X,C56X,C57X,C570,C571,C572,C573,C574,C577,C578,C579,C58X,C60X,C600,C601,C602,C608,C609,C61X,C62X,C620,C621,C629,C63X,C630,C631,C632,C637,C638,C639,C64X,C65X,C66X,C67X,C670,C671,C672,C673,C674,C675,C676,C677,C678,C679,C68X,C680,C681,C688,C689,C69X,C690,C691,C692,C693,C694,C695,C696,C698,C699,C73X,C74X,C740,C741,C749,C75X,C750,C751,C752,C753,C754,C755,C758,C759,C76X,C760,C761,C762,C763,C764,C765,C767,C768,C97X |
| Rheumatoid arthritis/ collagen vascular diseases | L940,L941,L943,M05X,M06X,M08X,M120,M123,M30X,M310,M311,M312,M313,M32X,M33X,M34X,M35X,M45X,M461,M468,M469 |
| Coagulopathy | D65X,D66X,D67X,D68X,D691,D693,D694,D695,D696 |
| Obesity | E66X |
| Weight loss | E40X,E41X,E42X,E43X,E440,E441,E45X,E46X,R634,R64X |
| Fluid and electrolyte disorders | E222,E86X,E87X |
| Blood loss anemia | D500 |
| Deficiency anemia | D508,D509,D51X,D52X,D53X |
| Alcohol abuse | F10X,E52X,G621,I426,K292,K700,K703,K709,T51X,Z502,Z714,Z721 |
| Drug abuse | F11X,F12X,F13X,F14X,F15X,F16X,F18X,F19X,Z715,Z722 |
| Psychoses | F20X,F220,F228,F229,F230,F233,F238,F239,F24X,F250,F251,F252,F258,F259,F28X,F29X,F302,F312,F315 |
| Depression | F204,F313,F314,F315,F32X,F33X,F341,F412,F432 |
| Dementia | F00X,F000,F001,F002,F009,F01X,F010,F011,F012,F013,F018,F019,F02X,F020,F021,F022,F023,F024,F028,F03X |
