# Supplementary material for: Variation in rates of post-operative oncological treatment for patients with glioblastoma in England: a comprehensive multi-year National cohort study from the GlioCova project
Source: J Neurooncol. 2026 Apr 25;177(3):126. doi: 10.1007/s11060-026-05440-7 (PMC13110236; doi:10.1007/s11060-026-05440-7)
Supplement: Supplementary file 2 — Supplementary Material 2 [file 11060_2026_5440_MOESM2_ESM.docx]

# Online Resource 2

Scores for modified Elixhauer Comorbidity Index variables using all brain/spinal tumour patient admissions between 2012-2019 based on English HES data. A higher score indicates that this comorbidity is linked with higher log odds of in-hospital mortality (e.g. a score of 2 is associated with a double increase in the log odds of mortality)

| **Comorbidity variable** | **Gliocova HES-based score 2013-2018,** **all CNS tumour inpatients** |
| --- | --- |
| **Alcohol abuse** | -6 |
| **Cardiac arrhytmias** | 2 |
| **Coagulopathy** | 2 |
| **Deficiency anemia** | -3 |
| **Depression** | -6 |
| **Diabetes uncomplicated** | 1 |
| **Drug abuse** | -6 |
| **Dementia** | 3 |
| **Fluid and electrolyte disorders** | 3 |
| **Hypothyroidism** | -4 |
| **Liver disease** | -2 |
| **Psychoses** | -3 |
| **Rheumatoid arthritis -collagen vascular diseases** | -5 |
| **Solid tumor - without metastasis** | 1 |
| **Weight loss** | -3 |
| **Blood loss anemia** | 0 |
| **Chronic pulmonary disease** | 0 |
| **Congestive heart failure** | 0 |
| **Diabetes complicated** | 0 |
| **Hypertension uncomplicated** | 0 |
| **Lymphoma** | 0 |
| **Other - neurological disorders** | 0 |
| **Paralysis** | 0 |
| **Peptic ulcer disease - excluding bleeding** | 0 |
| **Peripheral vascular disorders** | 0 |
| **Pulmonary circulation disorders** | 0 |
| **Renal failure** | 0 |
| **Valvular disease** | 0 |
| **Metastatic cancer** | 0 |
